# Effects of a Calcium Bentonite Clay in Diets Containing Aflatoxin when Measuring Liver Residues of Aflatoxin B_1_ in Starter Broiler Chicks

**DOI:** 10.3390/toxins7093455

**Published:** 2015-08-26

**Authors:** Justin Fowler, Wei Li, Christopher Bailey

**Affiliations:** 1 Department of Poultry Science, the University of Georgia, Athens, GA 30602, USA; 2 Office of the Texas State Chemist, College Station, TX 77843, USA; E-Mail: wei@otsc.tamu.edu; 3 Department of Poultry Science, Texas A&M University, College Station, TX 77843, USA; E-Mail: chris.bailey@ag.tamu.edu

**Keywords:** adsorbent, aflatoxin, bentonite, chicken, liver, residue

## Abstract

Research has shown success using clay-based binders to adsorb aflatoxin in animal feeds; however, no adsorbent has been approved for the prevention or treatment of aflatoxicosis. In this study, growth and relative organ weights were evaluated along with a residue analysis for aflatoxin B_1_ in liver tissue collected from broiler chickens consuming dietary aflatoxin (0, 600, 1200, and 1800 µg/kg) both with and without 0.2% of a calcium bentonite clay additive (TX4). After one week, only the combined measure of a broiler productivity index was significantly affected by 1800 µg/kg aflatoxin. However, once birds had consumed treatment diets for two weeks, body weights and relative kidney weights were affected by the lowest concentration. Then, during the third week, body weights, feed conversion, and the productivity index were affected by the 600 µg/kg level. Results also showed that 0.2% TX4 was effective at reducing the accumulation of aflatoxin B_1_ residues in the liver and improving livability in birds fed aflatoxin. The time required to clear all residues from the liver was less than one week. With evidence that the liver’s ability to process aflatoxin becomes relatively efficient within three weeks, this would imply that an alternative strategy for handling aflatoxin contamination in feed could be to allow a short, punctuated exposure to a higher level, so long as that exposure is followed by at least a week of a withdrawal period on a clean diet free of aflatoxin.

## 1. Introduction

Aflatoxins, which are secondary metabolites produced by the *Aspergillus flavus* and *parasiticus* species of mold, are among the most potent natural carcinogens. No animal species is immune to the toxic effects of consuming food contaminated with aflatoxin and *Aspergillus* species are known to infect important agricultural crops. Therefore, in cases where preventative management strategies in the field have failed to prevent *Aspergillus* species from producing aflatoxin in a crop, poultry producers require cost-effective methods of detoxifying potentially contaminated grain.

The use of clay-based adsorbents has proved effective at reducing the toxic effects of aflatoxin-contamination in animal feeds [1,2,3,4]. Specifically, bentonite clays are adsorbent aluminum silicates that are capable of adsorbing aflatoxin within the clay smectite layer, which allows any adhering aflatoxin to pass through the gastrointestinal tract unabsorbed. Calcium bentonite is a better absorbent of aflatoxin when compared to sodium bentonites because the calcium ions provide a better separation of the clay layers when compared to sodium ions [5]. Although these clay-based binders are generally recognized as safe (GRAS) to be used in diets for improved flowability, anti-caking, and pellet quality, and are approved by the European Commission for reduction of the contamination of feed by mycotoxins for all animal species [6], no adsorbent has been approved by the Food and Drug Administration for the prevention or treatment of aflatoxicosis. Concerns over the deposition of aflatoxin residues in edible tissues represent a part of the reason why. Aflatoxins can accumulate in the milk of dairy cattle, as well as in the muscle, liver, and eggs of poultry [7]. It is also possible for there to be detectable levels of aflatoxin residues without the animal’s growth or performance affected noticeably.

In this study, a residue analysis for aflatoxin B_1_ in samples of liver tissue collected at weekly intervals from broiler chickens consuming a range of dietary aflatoxin both with and without a calcium bentonite clay additive was conducted. These residue data were then compared to the more traditional measures of aflatoxicosis (growth rate, feed consumption, and the relative weights of the liver, kidney, and immune organs). The purpose being to gain both a better understanding of how aflatoxin affects broiler chickens, as well as to evaluate the efficacy of clay-based binders for their ability to prevent or treat aflatoxicosis.

## 2. Results

### 2.1. Broiler Performance and Relative Organ Weight

Aflatoxin had a significant effect on the broiler productivity index (which is a cumulative measure taking into account mortality, body weight, and feed conversion), suggesting that 1800 µg/kg was sufficient to reduce overall performance (Table 1) despite having no effect on body weight, feed conversion, or mortality independently. By the time birds had eaten treatment diets for two weeks, aflatoxin significantly reduced body weights as well as the productivity index beginning at the 600 µg/kg level. Also, cumulative mortality was significantly higher in the birds fed 1800 µg/kg when compared to those fed the 0 µg/kg control. The relative kidney weight began to be affected by aflatoxin during the second week, showing a significant increase at the 600 µg/kg level (Table 2). There was also a significant interaction between aflatoxin and clay on the kidney weights, in which those from the 1800 µg/kg treatment with the added TX4 were significantly smaller than those without clay. During week three (which was the final week that birds were on the aflatoxin-contaminated diets), aflatoxin had a significant main effect on all variables expect for the relative bursa of Fabricius weight. For body weight, cumulative feed-to-gain ratio, and productivity index, the 600 µg/kg level of aflatoxin impaired performance relative to 0 µg/kg. Cumulative mortality increased with the inclusion of 1800 µg/kg aflatoxin. The relative liver weights showed a significant increase in response to aflatoxin at 1200 µg/kg. Kidney and spleen weights were slightly more sensitive, showing changes at the 600 µg/kg level. This evident result was supported by statistical analysis that included an aflatoxin and weeks of age interaction term, which was highly significant (*p* < 0.001). With respect to including TX4, the only variable to show a main effect for 0.2% of the clay was cumulative mortality. Diets that included the calcium bentonite clay had significantly lower mortality than the control diets that had no clay added (Table 3).

**Table 1:** The effect of a calcium bentonite (TX4) included in the diet at 0.2% at ameliorating the effects of aflatoxin contamination on the performance of broilers during the first week on treatment diets.

Week 1						
Source of Variation	BW	FCR	PI	Mort	Rel Liver	Rel Kidney
0 µg/kg	168 ± 5	1.14 ± 0.02	212 ± 7 ^a^	0.0 ± 0.0	4.54 ± 0.21	0.83 ± 0.06
600 µg/kg	165 ± 3	1.11 ± 0.01	211 ± 6 ^a^	1.2 ± 1.2	4.61 ± 0.18	0.84 ± 0.06
1200 µg/kg	157 ± 7	1.12 ± 0.02	199 ± 10 ^ab^	1.2 ± 1.2	4.74 ± 0.17	1.01 ± 0.07
1800 µg/kg	151 ± 5	1.16 ± 0.02	182 ± 8 ^b^	2.6 ± 1.7	4.73 ± 0.16	0.89 ± 0.07
Control	163 ± 3	1.12 ± 0.02	204 ± 5	1.9 ± 1.0	4.66 ± 0.13	0.90 ± 0.05
TX4	159 ± 4	1.14 ± 0.01	198 ± 6	0.6 ± 0.6	4.65 ± 0.12	0.88 ± 0.04
**ANOVA**						
*Aflatoxin*	0.088	0.254	0.044	0.533	0.860	0.172
*Clay*	0.447	0.322	0.489	0.292	0.938	0.588
*Aflatoxin × Clay*	0.372	0.190	0.752	0.842	0.269	0.099

Data are presented as means ± SEM; ^a,b^ Means for main effects within a column lacking a common superscript differ (*p* ≤ 0.05); BW = Average body weight per bird (g); FCR = Cumulative feed-to-gain ratio; PI = Broiler productivity index (Livability [%] × Live weight [kg]/age [d]/FCR × 100); Mort = Cumulative mortality (%); Rel Liver = Liver weight as a % of BW; Rel Kidney = Kidney weight as a % of BW; *n* for clay type = 28; *n* for aflatoxin level = 14.

**Table 2:** The effect of a calcium bentonite (TX4) included in the diet at 0.2% at ameliorating the effects of aflatoxin contamination on the performance of broilers during the second week on treatment diets.

Week 2						
Source of Variation	BW	FCR	PI	Mort	Rel Liver	Rel Kidney
0 µg/kg	388 ± 9 ^a^	1.49 ± 0.02	185 ± 5 ^a^	1.2 ± 1.2 ^a^	3.74 ± 0.12	0.76 ± 0.04 ^a^
600 µg/kg	354 ± 9 ^b^	1.57 ± 0.05	162 ± 8 ^b^	1.2 ± 1.2 ^a^	3.66 ± 0.07	1.03 ± 0.07 ^b^
1200 µg/kg	326 ± 14 ^b^	1.54 ± 0.04	149 ± 9 ^b^	3.6 ± 2.0 ^ab^	4.14 ± 0.23	1.06 ± 0.08 ^b^
1800 µg/kg	293 ± 7 ^c^	1.62 ± 0.06	121 ± 7 ^c^	7.7 ± 2.4 ^b^	4.02 ± 0.22	1.10 ± 0.10 ^b^
Control	343 ± 10	1.56 ± 0.03	154 ± 7	3.7 ± 1.4	3.79 ± 0.11	1.00 ± 0.06
TX4	339 ± 10	1.54 ± 0.03	156 ± 7	3.0 ± 1.2	3.99 ± 0.14	0.97 ± 0.06
**ANOVA**						
*Aflatoxin*	<0.001	0.190	<0.001	0.047	0.154	0.002
*Clay*	0.805	0.533	0.733	0.651	0.276	0.409
*Aflatoxin × Clay*	0.256	0.251	0.799	0.502	0.233	0.001

Data are presented as means ± SEM; ^a,b,c^ Means for main effects within a column lacking a common superscript differ (*p* ≤ 0.05); BW = Average body weight per bird (g); FCR = Cumulative feed-to-gain ratio; PI = Broiler productivity index (Livability [%] × Live weight [kg]/age [d] / FCR × 100); Mort = Cumulative mortality (%); Rel Liver = Liver weight as a % of BW; Rel Kidney = Kidney weight as a % of BW; *n* for clay type = 28; *n* for aflatoxin level = 14.

**Table 3:** The effect of a calcium bentonite (TX4) included in the diet at 0.2% at ameliorating the effects of aflatoxin contamination on the performance of broilers during the third week on treatment diets.

Week 3								
Source of Variation	BW	FCR	PI	Mort	Rel Liver	Rel Kidney	Rel Spleen	Rel Bursa
0 µg/kg	764 ± 23 ^a^	1.72 ± 0.03 ^a^	210 ± 8 ^a^	1.2 ± 1.2 ^a^	3.23 ± 0.12 ^a^	0.69 ± 0.03 ^a^	0.11 ± 0.01 ^a^	0.21 ± 0.02
600 µg/kg	645 ± 17 ^b^	2.00 ± 0.12 ^bc^	56 ± 13 ^b^	4.8 ± 2.1 ^a^	3.85 ± 0.16 ^a^	0.98 ± 0.09 ^b^	0.16 ± 0.01 ^b^	0.20 ± 0.02
1200 µg/kg	581 ± 21 ^b^	1.83 ± 0.06 ^ab^	141 ± 7 ^b^	7.1 ± 2.9 ^ab^	4.87 ± 0.23 ^b^	1.30 ± 0.11 ^c^	0.19 ± 0.02 ^bc^	0.22 ± 0.01
1800 µg/kg	448 ± 40 ^c^	2.12 ± 0.10 ^c^	94 ± 11 ^c^	15.4 ± 4.8 ^b^	4.93 ± 0.32 ^b^	1.27 ± 0.12 ^c^	0.20 ± 0.02 ^c^	0.22 ± 0.01
Control	602 ± 34	1.95 ± 0.07	144 ± 12	9.9 ± 2.9 ^b^	4.04 ± 0.18	1.02 ± 0.08	0.16 ± 0.01	0.21 ± 0.01
TX4	623 ± 22	1.88 ± 0.06	158 ± 9	4.2 ± 1.4 ^a^	4.31 ± 0.22	1.07 ± 0.08	0.17 ± 0.01	0.21 ± 0.01
**ANOVA**								
*Aflatoxin*	<0.001	0.010	<0.001	0.008	<0.001	<0.001	<0.001	0.617
*Clay*	0.278	0.421	0.117	0.040	0.395	0.944	0.972	0.529
*Aflatoxin* × *Clay*	0.220	0.565	0.224	0.631	0.993	0.738	0.332	0.110

Data are presented as means ± SEM; ^a,b,c^ Means for main effects within a column lacking a common superscript differ (*p* ≤ 0.05); BW = Average body weight per bird (g); FCR = Cumulative feed-to-gain ratio; PI = Broiler productivity index (Livability [%] × Live weight [kg]/age [d]/FCR × 100); Mort = Cumulative mortality (%); Rel Liver = Liver weight as a % of BW; Rel Kidney = Kidney weight as a % of BW; Rel Spleen = Spleen weight as a % of BW; Rel Bursa = Bursa of Fabricius weight as a % of BW; *n* for clay type = 28; *n* for aflatoxin level = 14.

### 2.2. Liver Residues of Aflatoxin B_1_

After one week on the treatment diets, aflatoxin at 1200 µg/kg caused a significant increase in liver residues when compared to both the 0 and 600 µg/kg treatments. A further increase was seen in the 1800 µg/kg diet. There was a significant main effect for the inclusion of 0.2% of the TX4 clay, with clay providing an approximate 43.5% reduction in the accumulation of aflatoxin B_1_ residues in liver tissue. An interaction between aflatoxin and clay was also observed in the first week. The nature of the interaction was that aflatoxin did not significantly increase residues until the 1800 µg/kg dose, whereas they were increased by 1200 µg/kg when there was no clay present. After birds had been consuming contaminated diets for two weeks, the main effect for aflatoxin remained the same, with 1200 µg/kg causing a significant increase in liver residues when compared to the 0 µg/kg control. Though the trend in dose response remained the same, after two weeks, the 600 and 1200 µg/kg treatments were no longer significantly different from each other. During the final week on aflatoxin (week 3), the only birds that had detectable levels of aflatoxin B_1_ in the liver were those that had been fed the diet containing the highest level (1800 µg/kg) without the TX4 clay. One week on the 0 µg/kg control finisher diet was sufficient to remove any detectable residues from all of the treatments (Table 4).

**Table 4:** Liver residues of aflatoxin B_1_ (µg/kg), analyzed on a weekly basis. Three weeks were spent on aflatoxin-contaminated feed. During the fourth week, all birds consumed a non-contaminated finisher diet.

Source of Variation	Week 1	Week 2	Week 3	Week 4
0	ND ^a^	ND ^a^	ND	ND
600	0.53 ± 0.27 ^a^	1.51 ± 0.47 ^ab^	ND	ND
1200	1.24 ± 0.36 ^b^	2.21 ± 0.67 ^b^	ND	ND
1800	2.05 ± 0.29 ^c^	3.84 ± 0.63 ^c^	0.27 ± 0.27	ND
Control	1.15 ± 0.26 ^b^	2.18 ± 0.48	0.14 ± 0.14	ND
TX4	0.65 ± 0.23 ^a^	1.60 ± 0.43	ND	ND
**ANOVA**				
Aflatoxin	<0.001	<0.001	0.403	-
Clay	0.034	0.277	0.323	-
Aflatoxin × Clay	0.010	0.884	0.403	-

Data are presented as means ± SEM; ^a,b,c^ Means for main effects within a column lacking a common superscript differ (*p* ≤ 0.05); *n* for clay type = 20; *n* for aflatoxin level = 10.

## 3. Discussion

### 3.1. Broiler Performance and Relative Organ Weight

The relationship between a given dose of aflatoxin and the time spent consuming that concentration was evident in terms of the growth and performance parameters. It was first shown by Huff, *et al.* that young birds can be quite robust against relatively high levels of aflatoxin if they are fed for a short period of time. Chick body weights were not affected by aflatoxin levels as high as 5000 µg/kg during the first three days (250-fold higher than the FDA regulatory level of 20 µg/kg for immature poultry), but began to be reduced by 2500 µg/kg after six days and by 1250 µg/kg after 17 days [8]. Likewise, in this study, after one week on aflatoxin-contaminated diets, only the cumulative measure of the broiler productivity index was significantly affected by the highest concentration of dietary aflatoxin (1800 µg/kg). However, once birds had consumed their treatment diets for two weeks, cumulative mortality was also increased by the 1800 µg/kg treatment, and body weights were reduced by the 600 µg/kg treatment. Also, at week two, there began to be a significant effect of aflatoxin on the relative organ weights, with the kidney weights showing an increase when aflatoxin was present in the diet. The interaction between aflatoxin and clay suggested that 0.2% of TX4 was reducing kidney damage caused by the high 1800 µg/kg inclusion of aflatoxin. The emergence of overt toxic effects showed a relationship with the increased accumulation of aflatoxin B_1_ residues in the liver over the first two weeks, suggesting that as the toxin was building-up in the liver it was beginning to impair the birds’ metabolism and health.

Including the calcium bentonite binder only had a significant ameliorative effect on the total mortality at three weeks. This is in contrast to other reports using aluminosilicate or bentonite clays [1,2,3], which have shown significant protection against the effects of aflatoxin on growth rate, feed consumption, and relative organ weights. The aflatoxin created for these trials followed the protocol of Shotwell, *et al.* which inoculate rice with cultures of *Aspergillus* spp. [9], which results in a highly concentrated source to be added into the diet. In this study, as well as one done previously [10], aflatoxin was created using a more natural matrix by inoculating corn with *Aspergillus parasiticus*. This process produces a less concentrated source of aflatoxin, and the difference may help explain the lack of amelioration on growth performance and organ weights in this study.

### 3.2. Liver Residues

With respect to the liver residues of aflatoxin B_1_, when Fernández, *et al.* fed 2500 and 5000 µg/kg aflatoxin to broilers for 32 days, liver residues of aflatoxin B_1_ were only 0.16 and 0.15 µg/kg [11]. The authors attributed the low values to rapid liver metabolization of aflatoxin B_1_ into the other more water soluble forms destined for excretion from the body. In this study, only birds fed the highest level of aflatoxin (1800 µg/kg) without any adsorbent clay had detectable residues of aflatoxin B_1_ after three weeks on the diet. All other treatments had successfully processed all aflatoxin B_1_ out of the liver. In Hussain *et al.*, it was found that, overall, older birds have lower tissue residues of aflatoxin B_1_ when compared to younger birds, even when they were fed aflatoxin for the same amount of time [12]. Also, the authors found that the elimination of residues occurred earlier in older birds. This supports the finding in these data, which show that as the birds age, the liver’s metabolizing mechanisms for dealing with incoming aflatoxin B_1_ become more efficient and residues no longer accumulate. This study also found that the clearance time required to remove aflatoxin B_1_ residues from the liver to be quite short, less than one week on a clean corn diet.

Results from the first week showed that 0.2% TX4 was effective at reducing the accumulation of aflatoxin B_1_ residues in liver. Neeff *et al.*, fed 2500 µg/kg aflatoxin to broilers for three weeks and found significant increases liver concentrations of aflatoxin B_1_, as well as B_2_, G_1_, M_1_, and aflatoxicol (all of the forms of aflatoxin analyzed except for aflatoxin G_2_) relative to the 0 µg/kg control diet [13]. The amount of aflatoxin B_1_ in the liver, after the three weeks, was 8.32 µg/kg (total aflatoxins were 12.78 µg/kg). In that study, the liver residue analysis was the only variable for which the main effect for the clay additive showed complete amelioration by returning the aflatoxin-contaminated treatment to a level that was not significantly different than the aflatoxin-free control diet. The results from this study (during the first week) agree with this, in that a significant clay effect (for 0.2% of TX4) was seen only in the liver residues. Also, during the third week, birds from the 1800 µg/kg treatment with no added clay treatment had detectable levels of aflatoxin B_1_ in the liver, whereas the treatment with the added TX4 did not. However, after the first week, liver residue data were not any more sensitive in evaluating aflatoxin or clay main effects when compared to the traditional measures of growth performance and relative organ weights. For those parameters, the TX4 clay was effective at improving livability in birds exposed to dietary aflatoxin for three weeks.

## 4. Experimental Section

### 4.1. Diet Formulation

Aflatoxin for this trial was produced by inoculating yellow dent corn with live fungal cultures of *Aspergillus parasiticus*. Inoculated grain was then incubated for five days at 37 °C and then kept under green-house conditions for one week with daily mixing. Four mash basal diets were prepared by blending increasing concentrations of the aflatoxin-contaminated corn with non-contaminated corn (0%, 25%, 50% and 75% of the contaminated corn, respectively) into a basal soybean meal concentrate (Table 5). Each of the four aflatoxin diets were then sub-divided and one was blended with 0.2% of a calcium bentonite (TX4) and the other received no clay (as a Control). The TX4 clay was mined from the Gonzalez region of Texas and was selected for its purity (81.4% smectite) and high aflatoxin adsorption capacity (13.9% *w*/*w*) [14]. These eight diets were analyzed for total aflatoxins by the Office of the Texas State Chemist and were fed to the birds for three weeks. Aflatoxin-contaminated diets were found to contain approximately 0, 600, 1200 and 1800 µg/kg. Also, a single corn-soy finisher diet was formulated using only non-contaminated corn. This diet was fed to all birds as a “clearance” feed during the fourth week (Table 5).

### 4.2. Animal Husbandry and Tissue Collection

A total of 336 straight-run, Ross 308 broiler chicks obtained on the day of hatch were housed in three Petersime battery brooder units. Six birds were randomly selected for placement into one of the 56 pens. The eight treatments (*n* = 7) were assigned such that each treatment had equal representation throughout the rearing room. Also, initial weights per pen were controlled so as to be within ±10 grams of the total average flock weight of six chicks (30% of all birds were sampled to obtain an average flock weight per chick). All birds were allowed *ad libitum* access to feed and water throughout the study.

At weekly intervals, feed intake and body weight per pen were recorded and organs were collected and weighed from one bird selected randomly from each pen. For the first two weeks, the liver and kidney were removed from one bird per pen for the determination of the relative organ weight. For the final two weeks, the relative organ weight was determined for the liver, kidney, spleen, and bursa of Fabricius. At each weekly point, the collected livers were retained for an analysis of aflatoxin B_1_ residues in the tissue. All personnel handling the birds, feed, or fecal material from this study wore N95 particulate respirators (3M™, St. Paul, MN, USA), latex gloves, and spun-bonded polypropylene coveralls (VWR^®^; Radnor, PA, USA). All methods were approved by the Texas A&M Institutional Animal Care and Use Committee and the Institutional Biosafety Committee (IACUC 2014-0120).

**Table 5:** Composition and nutrient content of the treatment diets.

Ingredient	Starter Diet, week 1–3 (%)	Finisher Diet, week 4 (%)
Corn	58.4	67.7
Dehulled soybean meal	34.5	25.5
DL-methionine 98%	0.23	0.15
Lysine HCl	0.18	0.21
Animal/vegetable fat blend	2.76	2.93
Limestone	1.56	1.58
Mono-dicalcium phosphate	1.54	1.29
Salt	0.51	0.33
Trace mineral premix ^1^	0.05	0.05
Vitamin premix ^2^	0.25	0.25
**Calculated Nutrient Content (%)**		
Crude protein	22.0	18.4
ME (Kcal/kg)	3050	3150
Crude fat	5.32	5.73
Crude fiber	2.63	2.48
Calcium	0.95	0.89
AV Phosphate	0.71	0.38
Sodium	0.22	0.12
Methionine	0.56	0.44
Lysine	1.31	1.10

^1^ Trace mineral premix added at this rate yields (mg/kg): Zinc, 60.0; Manganese, 60.0; Iron, 60.0; Copper, 7.0; Iodine, 0.4; ^2^ Vitamin premix added at this rate yields (per kg): vitamin A, 11 kIU; vitamin D_3_, 3,850 IU; vitamin E, 45.8 IU; menadione, 1.5 mg; B_12_, 0.017 mg; biotin, 0.55 mg; thiamine, 2.93 mg; riboflavin, 5.96 mg; *d*-pantothenic acid, 20.17 mg; B_6_, 7.15 mg; niacin, 45.8 mg; folic acid, 1.74 mg; choline, 130.3 mg.

### 4.3. Liver Residues

For the four weekly time-points, a 7.5 g sample of defrosted liver tissue (from five birds per treatment) was homogenized in 30 mL of 80:20 methanol/water (*v*/*v*). These samples were centrifuged (1500× *g*) for 10 min and a 15 mL aliquot of the supernatant was collected and diluted in a 1:1 ratio with 20:80 methanol/water (*v*/*v*). An isotope-labelled internal standard of aflatoxin B_1_ (5 ng/mL) was added to each sample followed by vortexing. Samples were filtered through a 0.2 μm Whatman™ filter and subjected to an analysis using liquid chromatography tandem mass spectrometry to detect concentrations of aflatoxin B_1_ according to the methodology used by the Office of the Texas State Chemist [15].

### 4.4. Statistical Analysis

Data were analyzed as a two-way ANOVA for the main effects of clay, aflatoxin, and their interaction using the General Linear Model procedure of SPSS. Significant means (*p* ≤ 0.05) were separated using Duncan’s Multiple Range Tests. Data are presented as means ± SEM. Further, to confirm the effect of exposure to aflatoxin over time, the interaction between clay and aflatoxin with weeks of age was analyzed.

## 5. Conclusions

This study evaluated the effects of aflatoxin on growth, relative organ weights, and residue accumulation of aflatoxin B_1_ in the liver of growing broiler chickens and the ameliorative effects of including an adsorbent calcium bentonite clay in the diet. Also evaluated was the clearance time required for the birds to remove aflatoxin B_1_ from the liver once the diet was not contaminated with aflatoxins.

It was confirmed that dietary aflatoxin has negative effects on the growth, feed efficiency, and organ function of broiler chickens. Further, we showed that this effect is cumulative. Body weights, relative kidney weights, and mortality were not affected by aflatoxin until week 2, then there were further effects to feed conversion and the relative liver and spleen weights at week 3. Including a calcium bentonite adsorbent in the diet (at 0.2%) successfully reduced the accumulation of aflatoxin B_1_ residues in the liver during week 1, and improved livability over-all.

The clearance time required to remove aflatoxin residues from the liver was less than one week on a clean corn diet. Thus, the best strategy for managing aflatoxin contamination in feedstuffs such as corn may not necessarily be dilution throughout the lifetime of the animal, since the effects of lower doses will “build up” over time. Rather an alternative solution may be to allow for a short, punctuated exposure to higher doses, so long as that exposure is followed by at least a week of a withdrawal period on a clean diet free of aflatoxin.
